# Structure-based functional annotation of putative conserved proteins having lyase activity from *Haemophilus influenzae*

**DOI:** 10.1007/s13205-014-0231-z

**Published:** 2014-06-17

**Authors:** Mohd. Shahbaaz, Faizan Ahmad, Md. Imtaiyaz Hassan

**Affiliations:** 1 Department of Computer Science, Jamia Millia Islamia, New Delhi, 110025 India; 2 Center for Interdisciplinary Research in Basic Sciences, Jamia Millia Islamia, Jamia Nagar, New Delhi, 110025 India

**Keywords:** *Haemophilus influenzae*, Hypothetical protein, Sequence analysis, Structure analysis, Function prediction, Gene annotation, Structure–function relationship

## Abstract

*Haemophilus influenzae* is a small pleomorphic Gram-negative bacteria which causes several chronic diseases, including bacteremia, meningitis, cellulitis, epiglottitis, septic arthritis, pneumonia, and empyema. Here we extensively analyzed the sequenced genome of *H. influenzae* strain Rd KW20 using protein family databases, protein structure prediction, pathways and genome context methods to assign a precise function to proteins whose functions are unknown. These proteins are termed as hypothetical proteins (HPs), for which no experimental information is available. Function prediction of these proteins would surely be supportive to precisely understand the biochemical pathways and mechanism of pathogenesis of *Haemophilus influenzae*. During the extensive analysis of *H. influenzae* genome, we found the presence of eight HPs showing lyase activity. Subsequently, we modeled and analyzed three-dimensional structure of all these HPs to determine their functions more precisely. We found these HPs possess cystathionine-β-synthase, cyclase, carboxymuconolactone decarboxylase, pseudouridine synthase A and C, D-tagatose-1,6-bisphosphate aldolase and aminodeoxychorismate lyase-like features, indicating their corresponding functions in the *H. influenzae*. Lyases are actively involved in the regulation of biosynthesis of various hormones, metabolic pathways, signal transduction, and DNA repair. Lyases are also considered as a key player for various biological processes. These enzymes are critically essential for the survival and pathogenesis of *H. influenzae* and, therefore, these enzymes may be considered as a potential target for structure-based rational drug design. Our structure–function relationship analysis will be useful to search and design potential lead molecules based on the structure of these lyases, for drug design and discovery.

**Electronic supplementary material:**

The online version of this article (doi:10.1007/s13205-014-0231-z) contains supplementary material, which is available to authorized users.

## Introduction

*Haemophilus influenzae* strain Rd KW20 was the first organism whose genome was successfully sequenced in 1995 by Craig Venter’s group (Fleischmann et al. 1995). The genome of *H. influenzae* contains 1,740 protein-coding genes, 2 transfer RNA genes, and 18 other RNA genes in a single circular chromosome of 1,830,140 base pairs (Fleischmann et al. 1995). This organism belongs to the family *Pasteurellaceae* (Christensen 2008; Kuhnert 2008), a non-typeable (NTHi) causing bacteremia and acute bacterial meningitis in infants, children and adults (Murphy and Sethi 1992; Sethi and Murphy 2001). *H. influenzae* is part of the normal nasopharyngeal flora in humans and is often coupled with otitis media, chronic bronchitis and community-acquired pneumonia (Apisarnthanarak and Mundy 2005; Eldika and Sethi 2006). *H. influenzae* is obligatory to human and requires β-nicotinamide adenine dinucleotide and heme for growth (Markel et al. 2007; Morton et al. 2004a). Due to its inability to produce iron-containing heme for the cytoplasmic enzymes, it uses multiple mechanisms to obtain heme (Stojiljkovic and Perkins-Balding 2002) that include various heme acquisition proteins like the heme utilization protein, Hup (Morton et al. 2004b) and heme-binding lipoprotein Hbp A (Morton et al. 2005). Mechanism for heme acquisition indicates a strict regulation of iron homeostasis in *H. influenzae,* and it is important for its survival and virulence (Morton et al. 2004a).

The sequenced genome of various organisms on comparative genomics analysis shows that a significant portion of the genes encodes “hypothetical proteins (HPs)”, i.e. functionally uncharacterized proteins but found in organisms (Galperin and Koonin 2004). HPs are predicted to be expressed from an open reading frame, but no experimental evidences are available for their function. The majority of HPs is believed to be a product of pseudogenes in human as well as of other organisms and constitute an extensive fraction of their proteomes (Desler et al. 2012; Galperin 2001). HPs have unique sequences and are essential determinants of species-specific phenotypic properties, such as pathogenicity in a given organism (Desler et al. 2012; Galperin 2001). These determinants are considered as a potent drug targets in pathogenic organisms (Kumar et al. 2014; Tsoka and Ouzounis 2000). Experimental characterization of these HPs in a model organism, such as *Escherichia coli*, *Saccharomyces cerevisiae, H. influenzae* etc., will be helpful in complete understanding of their biological systems at the molecular level (Nimrod et al. 2008; Park et al. 2012; Pidugu et al. 2009). The recent functional annotation of previously uncharacterized tRNA modification enzymes (Alexandrov et al. 2002; Jackman et al. 2003; Soma et al. 2003) of the deoxyxylulose pathway (Eisenreich et al. 2001, 2004), and of the central role of cyclic diguanylate in bacterial signaling (Galperin 2004; Jenal 2004) emphasizes the importance HP functional characterization.

A precise prediction of protein functions depends on accurate protein folding, which is revealed by their three-dimensional structure (Dobson 2003). Similarity in protein structure leads to similarity in biological function that cannot be predicted by sequence analysis alone, for the protein sequence is less conserved than the tertiary structure of a protein (Illergard et al. 2009). Available information on protein structure is useful for characterization of binding motifs and catalytic cores present in proteins (Shapiro and Harris 2000). Knowledge of protein structure is important for rational drug design that mainly relies on the structure of the target protein to narrow the searching of lead compound and for refinement of the compound (Capdeville et al. 2002; Klebe 2000). Hence, structure prediction of HPs may deliver some evidences about their biological function and help in the drug discovery of better therapeutics for treating diseases caused by *H. influenzae*.

Recently, we have annotated the function of HPs from *H. influenzae* (Shahbaaz et al. 2013). During extensive sequence analysis of HPs from *H. influenzae,* we predicted that eight HPs possess lyase activity as listed in Table 1. Since working on the structure-based drug design therefore we are looking for the novel therapeutic targets (Hassan et al. 2007a, b; Thakur et al. 2013; Thakur and Hassan 2011). Lyase enzymes are important for virulence and survival of pathogens in the host. These enzymes provide essential nutrients and are involved in modifying the local environment for the favorable growth (Bjornson 1984). Two examples of such enzymes are cystathionine β-lyase and isocitrate lyase. The formation and degradation of cystathionine cause the mobilization of sulfur from Cys, which is required for the biosynthesis of methionine in bacteria and is important for the virulence (Ejim et al. 2004). Similarly, the isocitrate lyase is an enzyme of the glyoxylate pathway (an anaplerotic pathway of the TCA cycle), which cleaves isocitrate to glyoxylate and succinate, which are potential drug targets for control of many human diseases caused by various pathogens (Britton et al. 2001). Thus, lyase enzymes are used for sequence and structure analysis for further study in this paper.

**Table 1:** List of hypothetical protein with lyase activity in *H. influenzae* strain Rd KW20

S. no.	Accession no	Gene ID	Protein product	Uniprot ID	Protein name
1	NC_000907.1	949660	NP_438613.1	P44717	HP HI0452
2	NC_000907.1	950684	NP_438775.1	P44782	HP HI0617
3	NC_000907.1	950339	NP_439586.2	P44197	HP HI1435
4	NC_000907.1	950454	NP_439740.1	P45267	HP HI1598
5	NC_000907.1	950030	NP_439212.1	Q57498	HP HI1053
6	NC_000907.1	949991	NP_439177.1	P44095	HP HI1016
7	NC_000907.1	950004	NP_439172.1	P44093	HP HI1011
8	NC_000907.1	950653	NP_438618.1	P44720	HP HI0457

## Materials and methods

### Sequence retrieval

The genome analysis of *H. influenzae* shows that 1,657 proteins are present in its proteome (http://www.ncbi.nlm.nih.gov/genome/). We have selected all 429 proteins (Table S1 and S2) with a name HP separately and perform rigorous sequence analyses using fasta sequences retrieved from UniProt (http://www.uniprot.org/) (Shahbaaz et al. 2013). Sequences of proteins having lyase-like activity were used for further structure prediction and analysis.

### Sub-cellular localization

To characterize a protein as a drug and/or vaccine target, sub-cellular localization is essentially important. We used sub-cellular localization tools, PSORTb (Yu et al. 2010b), PSLpred (Bhasin et al. 2005) and CELLO (Yu et al. 2006). SignalP 4.1 (Emanuelsson et al. 2007) was used for signal peptide prediction and SecretomeP (Bendtsen et al. 2005) for identifying protein involvement in non-classical secretory pathway. TMHMM (Krogh et al. 2001) and HMMTOP (Tusnady and Simon 2001) were used to predict the propensity of a protein to be a membrane protein.

### Sequence comparisons

We predicted the function of HPs using conserved sequence patterns in protein families (Chen and Jeong 2000). BLASTp (Altschul et al. 1990) and HHpred (Soding et al. 2005) based on hidden Markov models were used for remote protein homology detection against various protein databases such as PDB (Bernstein et al. 1978), SCOP (Hubbard et al. 1999), CATH, etc.

### Function prediction

The domain analysis allows more precise function prediction (Reid et al. 2007). We used various publicly available databases for functional annotation such as Pfam (Punta et al. 2012), PANTHER (Mi et al. 2005), SMART (Letunic et al. Letunic et al. 2012), SUPERFAMILY (Gough et al. 2001), CATH (Sillitoe et al. 2013), CDART (Geer et al. 2002), SYSTERS (Meinel et al. 2005), ProtoNet (Rappoport et al. 2012) and SVMProt (Cai et al. 2003). CDART and SMART were used for similarity search based on domain architecture and profiles rather than by direct sequence similarity. MOTIF (Kanehisa 1997) and InterProScan (Quevillon et al. 2005) help to identify signature sequence in a particular protein. The MEME suite (Bailey et al. 2009) has been used to perform motif-sequence database searching.

### Virulence factor analysis

Virulence factors are considered as potential drug/vaccine targets (Baron and Coombes 2007). We used VICMpred (Saha and Raghava 2006) and Virulentpred (Garg and Gupta 2008) for identifying potential virulence factors. Both are SVM-based methods used to predict bacterial virulence factors from protein sequences with a significant accuracy.

### Functional protein association networks

To understand the role of a protein in certain biological pathways, protein–protein interaction analysis is very useful. Here, we used STRING (version-9.05) (Szklarczyk et al. 2011) to predict protein interaction partners of HPs. The interactions include direct (physical) and indirect (functional) associations, experimental or co-expression.

### Structure prediction

Three-dimensional structure of proteins P44197 and P44720 was predicted using the homology modeling (Marti-Renom et al. 2000) on MODELLER module present in the Discovery Studio 3.5 (Accelrys 2013). We have identified templates using sequence similarity search methods PSI-BLAST (Altschul et al. 1997) and found pseudouridine synthases RluC (Mizutani et al. 2004) and aminodeoxychorismate lyase from *Escherichia coli* (PDB id: 2RIF) as a suitable template for P44197 and P44720, respectively. The template and query sequences were aligned and finally used for modeling protein structure in MODELLER (Eswar et al. 2007).

Since we do not find any suitable template for other HPs with lyase activity, MODELLER has no role in structure prediction of such proteins. We used threading or fold recognition and ab initio modeling protocol for predicting the structure of such proteins. The threading or fold recognition (Xu et al. 2008) methods were applied for aligning the primary sequence with those proteins present in the Protein Data Bank (PDB) with similar folds. It uses sequence–structure alignment approaches for modeling. We used Sparksx server (Yang et al. 2011) for predicting the structure of P44782, P45267 and Q57498, which uses the SPINE X server for predicting secondary structure, torsion angles and solvent accessibility with higher accuracy. For protein P44093, we used RaptorX (Peng and Xu 2011), as sparksx models are not found suitable for further studies which use NEFF to measure the amount of information in the sequence profile of a protein. However, sparksx along with PSI-BLAST and HHpred is considered to be the best server for predicting the hard targets in Critical Assessment of protein Structure Prediction (CASP) proceedings (Kryshtafovych et al. 2011).

ROBETTA server (Kim et al. 2004) was used for structure prediction of HP P44717, which uses ab initio or de novo methods to predict the structure of proteins whose structural analogs do not exist in the PDB or could not be successfully identified by threading. ROBETTA server generates structure from scratch, since it uses a new alignment method, called K*Sync, to align the query sequence onto the parent structure. Then it models variable regions by allowing them to explore conformational space with fragments in a fashion similar to the de novo protocol in context of the template. When no structural homolog is available, domains are modeled with the Rosetta de novo protocol (Misura et al. 2006), which allows the full length of the domain to explore conformational space via fragment insertion, producing a large decoy ensemble from which the final models are selected.

We used I-TASSER (Roy et al. 2010) for predicting the structure of HP P44095 that first generates three-dimensional (3D) atomic models from multiple threading alignments and iterative structural assembly simulations. Using the structural matching of the 3D models with other known proteins it inferred function of the protein and the output contains full-length secondary and tertiary structure predictions, and functional annotations on ligand-binding sites, Enzyme Commission numbers and Gene Ontology terms in the results (Roy et al. 2010). Since the unavailability of proper prediction, we used the COACH (Yang et al. 2013) for protein–ligand-binding site prediction and COFACTOR (Roy et al. 2012) for structure-based biological function annotation of HP, for proper function analysis of P44095.

Predicted models were further refined using a side-chain refinement protocol of Discovery Studio 3.5, which uses force fields such as CHARMM (Brooks et al. 2009) and SCWRL4 (Krivov et al. 2009), and predicts positions of the side chains which are used for refinement of predicted protein structures. The final models were further validated by PROCHECK suite (Laskowski et al. 1996), a module of SAVES (Structural Analysis and Verification Server).

### Structure analysis

Three-dimensional structure of homologous proteins often remains more conserved than their sequence (Chothia and Lesk 1986). Structural similarities are more reliable than that of sequences for grouping together distant homologues (Taylor and Orengo 1989). Three-dimensional structure of all models was analyzed by various servers for function prediction. Identifying the functional region of protein is an important step towards characterizing its molecular function. We used POCASA (Yu et al. 2010a), Pocket-Finder (Laurie and Jackson 2005) and firestar server (Lopez et al. 2007) for prediction of functionally important residues in HP and the PPM server for calculating rotational as well as translational positions of transmembrane and peripheral proteins in cell membranes (Lomize et al. 2012). It can be applied to newly determine experimental protein structures or theoretical models. Since structural motifs are associated with catalytic functions, motif finding is also essential for precise function prediction (Singh and Saha 2003). We used ProFunc (Laskowski et al. 2005) web server for predicting the function of proteins from its atomic coordinates based on identification of functional motifs. DALI server (Holm and Rosenstrom 2010) was used for structure comparison and fold similarity search. PyMOL (DeLano 2002), a molecular graphics system, is used for visualization of protein structure.

## Results and discussion

After the extensive sequence and structure analysis, we found eight HPs with characteristic function, namely, cystathionine-β-synthase, cyclase family protein, carboxymuconolactone decarboxylase, pseudouridine synthase A and C, tagatose-1,6-bisphosphate aldolase and aminodeoxychorismate lyase proteins (Table 2). We successfully predicted and analyzed the three-dimensional structure of HPs with lyase activity, using various computational tools. Final models were further validated to check their stereo-chemical parameters such as bond angle and bond length. All eight models P44782, P44717, P45267, Q57498, P44197, P44095, P44720 and P44093 show significant validation score on SAVES server (http://nihServer.mbi.ucla.edu/SAVES/). Structure analysis helped us to predict the function of each HP precisely (Table 3). Detailed structural analysis outcomes for each protein are described here, separately.

**Table 2:** List of sequence-based predicted function of HPs with lyase activity and motif discovered using MEME of *H. influenzae* strain Rd KW20

S. no.	Cluster^a^	Uniprot ID	MEME results							Consensus^b^ function
			Motif 1		Motif 2		Motif 3		MAST function prediction	
			Start	Site	Start	Site	Start	Site		
1	Cluster 118	P44717	262	GYIESH	199	TQEHYL	405	YGKYKF	UPF0053 protein	Cystathionine-beta-synthase
2	Cluster 187	P44782	152	VKLTPVTGRSHQLRLHMLALGHPILGDKFY	56	FCEPAHRLDMATSGIIVFALSKAADRELKRQFREREPKKHYQAIVWGH	18	YQDNHLCVVNKPSG	Ribosomal large subunit pseudouridine synthase A	RNA pseudouridylate synthase rluA (lyase)
3	Cluster 187	P44197	152	VKLIPHTGRKHXLRXHMKHVFHPIXGDTQY	46	HVFPIHRLDRPTSGVLLFALSSEIANLMCEQFEQKYVQKSYLAVVRGY	6	YQDGFLVAVNKPAG	tRNA pseudouridine synthase C	Ribosomal large subunit pseudouridine synthase C rluC
4	Cluster 114	P45267	335	RVYFERM	14	AISPQI	253	FSQDFM	No result	CYTH-like adenylate cyclase
5	Cluster 196	Q57498	1	MFTDWK	59	TRCESC	21	KQYPKM	No result	Carboxymuconolactone decarboxylase
6	Cluster 131	P44095	128	GIWFPCTW	21	PNHCGTHM	11	TPFSSF	No result	Cyclase family protein
7	Cluster 45	P44093	68	WLKENGCTQFYFKYCST	281	IHNENYIE	151	NLMRLM	No result	D-tagatose-1,6-bisphosphate aldolase
8	Cluster 89	P44720	315	DGSGGH	123	WRKDLENAPH	338	RWYRSQ	UPF0755 protein	Aminodeoxychorismate lyase

^a^CLUSS predicted clusters

^b^Consensus result form on the basis of values present in supplementary Table S2 & S3

**Table 3:** List of structure-based predicted function and validation of HP with lyase activity in *H. influenzae* strain Rd KW20

S. no.	Uniprot ID	Template	Identity (%)	RMSD	Ramachandran	Proposed function
1	P44717 (robetta model)	Magnesium and cobalt efflux protein CorC, 4HG0 CorC/HlyC transporter-associated domain of a CBS domain protein, 2PLS	23 60	3.400 0.598	99 % (91.7 % core 6.5 % allow 0.8 % gener 1.0 % disall)	CBS domain associated CorC/HlyC transporter
2	P44782 (sparksx model)	Template-pseudouridine synthase RluA, 2I82	60	0.174	99.5 % (91.1 % core 8.4 % allow 0.0 % gener 0.5 % disall)	Pseudouridine synthase RluA
3	P44197 (Modeller structure)	Pseudouridine synthase RluC, 1XPI Pseudouridine synthase RluC, 1V9K	34 33	0.379 0.334	99.5 % (88.2 % core 8.8 % allow 2.5 % gener 0.5 % disall)	Pseudouridine synthase RluC
4	P45267 (Sparksx model)	Putative adenylate cyclase, 2GFG	39	0.918	98.8 % (88.9 % core 9.6 % allow 0.3 % gener 1.2 % disall)	Adenylate cyclase
5	Q57498 (Sparksx model)	Template—Carboxymuconolactone decarboxylase family protein, 1VKE	31	0.300	99 % (94.2 % core 2.9 % allow 1.9 % gener 1.0 % disall)	Carboxymuconolactone decarboxylase
6	P44095 (I-TASSERmodel)	Manganese dependent isatin hydrolase, 4J0N (A) Metal-dependent hydrolase with cyclase activity, 1R61	54 27	2.752 1.139	98.3 % (88.4 % core 9.1 % allow 0.8 % gener 1.7 % disall)	Metal-dependent hydrolase with cyclase activity
7	P44093 (Raptorx model)	Putative tRNA synthase, 3DQQ	56	0.215	99.7 % (93.1 % core 6.6 % allow 0.0 % gener 0.3 % disall)	Putative tRNA synthase
8	P44720 (Modeller)	Predicted aminodeoxychorismate lyase protein, 2R1F	49	0.378	99.7 % (89.8 % core 8.3 % allow 1.6 % gener 0.3 % disall)	Aminodeoxychorismate lyase protein

### HP P44717

Sub-cellular localization prediction of HP P44717 indicates that this HP is localized in the cytoplasmic membrane. Signal peptide prediction suggests the presence of a signal peptide, and indicates that its translocation occurs through non-classical secretory pathway. HMMTOP (Tusnady and Simon 2001) and TMHMM (Krogh et al. 2001) analyses suggest that HP P44717 consists of four transmembrane helix, and may work as a transporter protein (Table S3). Similarity search revealed its high closeness to the cystathionine-β-synthase (CBS) domain protein. HHpred (Soding et al. 2005) also suggests high similarity with magnesium and cobalt efflux protein CorC (PDB ID: 4HG0) and CBS domain protein (PDB ID: 3LHH) (Table S4). Sequence analyses of P44717 suggest that P44717 is comprised of two domains, CorC/HlyC and CBS domain. By applying the clustering algorithms, we analyzed that HP P44717 is a member of cluster 143846 (CBS domain protein) of SYSTERS (Meinel et al. 2005) and cluster 4144744 (Transporter-associated region) of ProtoNet (Rappoport et al. 2012), and a member of transmembrane family protein. Sequence-based motifs are discovered using InterProScan (Quevillon et al. 2005) and MOTIF tools (Kanehisa 1997). We further observed that its sequence possesses cystathionine-β-synthase motif and CBS domain motif. Another motif discovery search tool, MEME suite (Bailey et al. 2009) has detected three motifs, namely, 262′-GYIESH, 199′-TQEHYL and 405′-YGKYKF (Table 2). Based on all these observations, we suggest that HP P44717 may function as cystathionine-β-synthase and present in the inner plasma membrane to work as a transporter for various inorganic salts.

The virulence factor analysis shows that HP P44717 is a non-virulent protein. We further analyzed the functional protein association networks of HP P44717 using an online server STRING. This protein is showing close interaction with thymidylate kinase, 16S ribosomal RNA methyltransferase, virulence-associated protein D, hemolysin, DNA repair protein and GTP-binding protein (Figure S1).

**Fig. 1:**
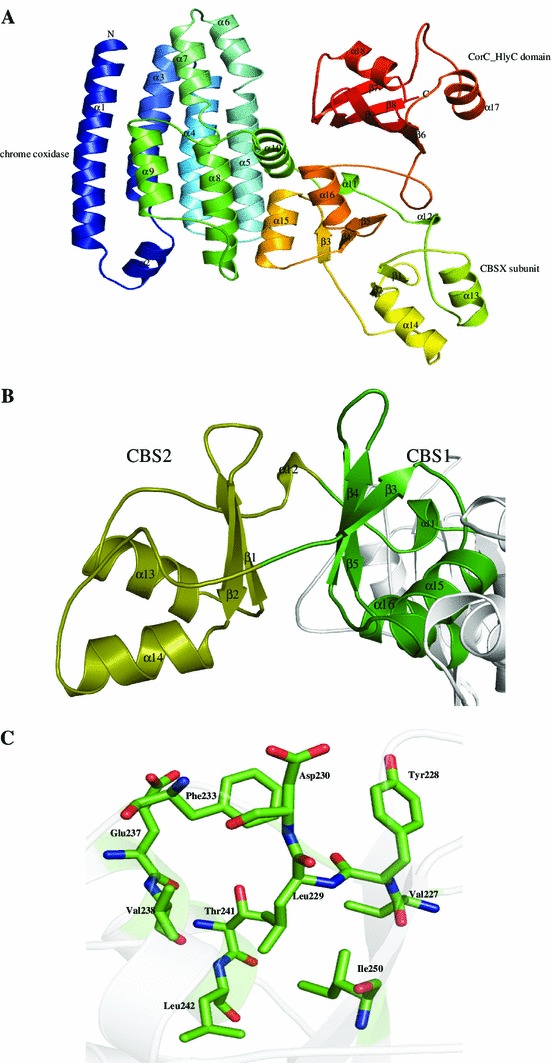
Representation of model structure of HP P44717. **a** Showing CBS, CorC_HlyC and cytochrome c oxidase domains. **b** Diagram showing the monomer of CBSX with CBS1 and CBS2 domain. **c** Residues present in the active site pocket are illustrated in stick

We predicted three-dimensional structure of P44717 using online server Robetta (Kim et al. 2004). The predicted structure was energy minimized, and validated using online server SAVES showing 99 % of residues in the allowed region of the Ramachandran plot (Hooft et al. 1997; Ramachandran et al. 1963). The root-mean-square deviation (rmsd) of the model with respect to the templates 4HG0 and 2PLS was 3.400 and 0.598 Å^2^, respectively (Table 3), indicating close functionality. Structure comparison and analysis further revealed that HP P44717 shows the presence of three functional domains, namely, CBS domain, cytochrome *c* oxidase polypeptide and CorC_HlyC domain (Fig. 1a). The overall structure of P44717 is comprised of 18 α-helices and nine β-strands including a CBSX subunit that contains two conserved CBS domains which form a pair facing each other, and are connected by two fundamental β-strands (β1–β2 and β3–β5) and bordered by six α-helices. The presence of helix α14 and helix α16 is the main feature of identifying dimeric CBS domain proteins, and both helices are critical for the dimer assembly of CBSX subunit (Fig. 1b). The CorC_HlyC domain contains four β-strands (β6–β9) arranged as anti-parallel β-strand and two α-helices (α17 and α18) that contain binding sites for metal ion binding to amino acids Ile413, Asp414, Thr415 (β8) and Asp421 (β9) (Figure S2 A and B). We also detected an AMP-binding site in P44717 structure at Val227 using Firestar server analysis (Figure S2 C). The active site cavity was predicted by Pocket-Finder (Laurie and Jackson 2005) showing residues Val227, Tyr228, Leu229, Asp230, Phe233, Glu237, Val238, Thr241, Leu242 and Ile250 which are presumably responsible for the lyase activity (Fig. 1c).

In order to find structurally similar proteins in the PDB related to the P44717, we used DALI server (Holm and Rosenstrom 2010). The results clearly indicate a significant similarity of the CBS domain containing proteins such as magnesium and cobalt efflux protein (*Z* score = 13.8), hemolysin-like protein (*Z* score = 13.4), etc., which are among the top hits. In each case, residues at positions 80–150 in the sequence show a close resemblance with rmsd of 0.8–3.2 Å^2^, despite a sequence similarity of 34 % in the aligned region. We also found a close structural similarity to the magnesium and cobalt efflux protein. Furthermore, ProFunc server (Laskowski et al. 2005) was used to predict the function of the HP on the basis of sequence and structure comparisons. We found 11 motifs in the InterPro database (Hunter et al. 2011) with CorC_HlyC, CBS and hemolysin related sequence motifs. There are two significant ligand-binding templates also present in the P44717 structure. All these analyses strongly suggest that HP P44717 contains CBS domain and it has CorC/HlyC transporter function (Table 3).

The CBS domain, also known as ‘Bateman domain’, is a conserved domain present in prokaryotes and eukaryotes (Ignoul and Eggermont 2005). Structure analyses of bacterial CBS domains show that two CBS domains form an intramolecular dimeric structure (CBS pair). Human hereditary diseases, such as hypertrophic cardiomyopathy, homocystinuria, myotonia congenital, retinitis pigmentosa, etc., can be caused by mutations in CBS domains of cystathionine-β-synthase, inosine 5′-monophosphate dehydrogenase, AMP kinase, and chloride channels, respectively, which also affect multimerization and sorting of proteins, channel gating, and ligand binding (Bateman 1997). Recent experiments show that CBS domains can bind adenosine-containing ligands such as ATP, AMP, or *S*-adenosylmethionine, which indicate that CBS domains may function as sensors of intracellular metabolites (Bateman 1997).

### HP P44782

HP P44782 is localized in the cytoplasm and lacks signal peptide, therefore, it is not secreted (Table S3). The sequence analysis showed a significant similarity of HP P44782 with 23S rRNA/tRNA pseudouridine synthase A (Table S1 and S2), which was further confirmed by domain annotation and cluster analysis using online tool SYSTERS and ProtoNet. Furthermore, a pseudouridine synthase, RsuA/RluB/C/D/E/F motif, and pseudouridine synthase, the RluA motif, are present in the sequence of HP P44782. MEME suite has further confirmed three distinct motifs, namely, 152′-VKLTPVTGRSHQLRLHMLALGHPILGDKFY, 56′-FCEPAHRLDMATSGIIVFALSKAADRELKRQFREREPKKHYQAIVWGH, 18′-YQDNHLCVVNKPSG (Table 2), an indication of ribosomal large subunit pseudouridine synthase A. Interacting partners of HP P44782 are ATP-dependent helicase, tRNA pseudouridine synthase B, tRNA pseudouridine synthase A, tRNA-dihydrouridine synthase A, glp protein, lipoprotein signal peptidase, glycerol-3-phosphate regulon repressor, 16S pseudouridylate 516 synthase, *S*-adenosyl-l-methionine-dependent methyltransferase (Figure S1) indicating its role in the pseudouridine synthesis.

**Fig. 2:**
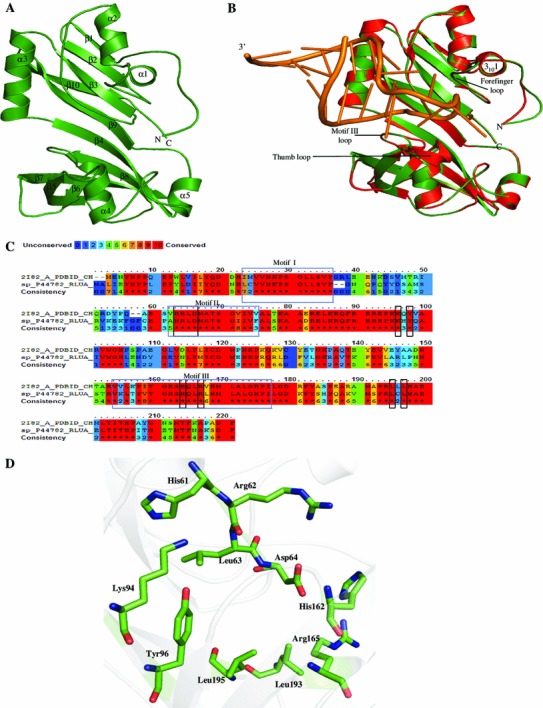
Representation of model structure of HP P44782. **a** Overall structure is represented in cartoon. **b** Superposition of the RluA–RNA complex (PDB ID: 2I82; *red*) with free RluA (P44782; *dark green*). The forefinger and thumb loops that “grasp” the RNA are indicated with motif III loop, and contrast the absence of the thumb loop in P44782 structure. **c** Multiple sequence alignment of P44782 and Rlu A (PDB ID: 2I82) with *red* color indicates residues that are conserved in two proteins. Motifs I, II, and III are as defined by Koonin (1996) highlighted in *blue boxes* and conserved active site residues in *black boxes*. **d** Predicted active site residues shown in stick

Sparksx predicted the structure of P44782 using ribosomal large subunit pseudouridine synthase A (PDBID: 2I82), a lyase, as a template. The final model was validated on PROCHECK (Laskowski et al. 1996) which shows that 99.5 % residues are in the allowed region of Ramachandran plot. Model structure showed rmsd of 0.174 Å^2^ from the template. The overall structure of P44782 is elongated and is comprised of mixed α/β fold (Fig. 2a, b). The structure is a conserved Ψ synthase fold, with an eight-stranded β sheet core, and additional strand extends the core β sheet which is also encircled by helices and loops on one face. ASL-RNA binds with its helical axis nearly parallel to the plane of the β sheet core of the predicted P44782 model (Fig. 2b). We found three motifs in sequence of HP P44782 (Fig. 2c) (Koonin 1996). Motif II is the part of the active site while motif I plays an architectural role by supporting motif II (Hoang and Ferre-D’Amare 2001, 2004). The superimposition of structures of P44782 and pseudouridine synthase RluA (PDB code: 2I82) indicates that C-terminal side of the motif I forms a projection that packs against minor groove face of the anticodon loop. N-terminal half of motif III (conserved among Ψ synthases of the RluA and RsuA families) interacts with the RNA backbone on the 5′- side of the ASL (Fig. 2b). The “thumb loop” and “forefinger loop” are found in pseudouridine synthase RluA (Hoang et al. 2006). Structure alignment showed the presence of forefinger loop but not the thumb loop in the HP P44782. The forefinger loop makes RluA enzyme bind with the minor groove of the substrate RNA. Previously determined crystal structure shows that families of pseudouridine synthases have universal conservation in the active sites as an aspartate, a basic residue, and a tyrosine (Del Campo et al. 2004; Koonin 1996; Ramamurthy et al. 1999). Amino acids corresponding to Asp64, Tyr96, and Arg165 are conserved in all ψ synthases, and the active site of RluA (Hoang et al. 2006) is comprised of residues His 61, Arg 62, Leu 63, Asp64, Lys94, Tyr96, His162, Arg165, Leu193 and Leu195 (Fig. 2c, d).

Structure similarity search using DALI server provides many significant hits for P44782 having ribosomal large subunit pseudouridine synthase activity, with ribosomal large subunit pseudouridine synthase C (*Z* score = 27.3), ribosomal large subunit pseudouridine synthase F (*Z* score = 12.8), ribosomal large subunit pseudouridine synthase E (*Z* score = 12.7), etc., among the top hits. We observe an rmsd of 0.4–3.0 Å^2^ for residues 180–250 in each match with 60 % sequence identity. Structure of P44782 was further analyzed using ProFunc server showing five sequence pseudouridine synthase motif and six significant ligand-binding templates. The ProFunc and Dali server searches clearly indicate the presence of pseudouridylate synthase activity in the HP P44782 (Table S4).

The isomerization of uridine to pseudouridine (Ψ) within RNA, discovered often in highly conserved locations in ribosomal and transfer RNA, is a ubiquitous process reported to be present in early stages in the evolution of life (Ofengand et al. 2001). Pseudouridine (Ψ) synthases are enzymes that catalyze site-specific isomerization of uridine residues in cellular RNAs. Ψ synthases can be classified into five families, namely, RluA, RsuA, TruA, TruB, and TruD on the basis of amino acid sequence (Kaya and Ofengand 2003), and structure analyses revealed that in spite of minimal sequence similarity, the cores of all Ψ synthases adopt the same fold (Ferre-D’Amare 2003; Foster et al. 2000; Sivaraman et al. 2002) and enzymatic core includes an aspartate residue that is conserved among all five families of Ψ synthases. On site-directed mutagenesis this Asp was found to be essential for catalytic activity of such enzyme (Conrad et al. 1999; Ramamurthy et al. 1999).

### HP P44197

HP P44197 is localized in the cytoplasm, and lacks signal peptide and transmembrane helix (Table S3). This protein showed high sequence similarity to tRNA pseudouridine synthase C. The domain annotation shows that P44197 is a member of pseudouridine synthase RsuA/RluD family, showing pseudouridylate synthase activity (Table S2). Inter ProScan and MOTIF tools further confirmed a pseudouridine synthase motif in the HP P44197 (Table 2). Predicted functional partners of HP P44197 are exodeoxyribonuclease VII small subunit, exodeoxyribonuclease VII small subunit, 1-deoxy-d-xylulose-5-phosphate synthase, aspartate-semialdehyde dehydrogenase, tRNA pseudouridine synthase B, and tRNA pseudouridine synthase A which provide an insight of pseudouridine synthase activity for this HP (Figure S1).

**Fig. 3:**
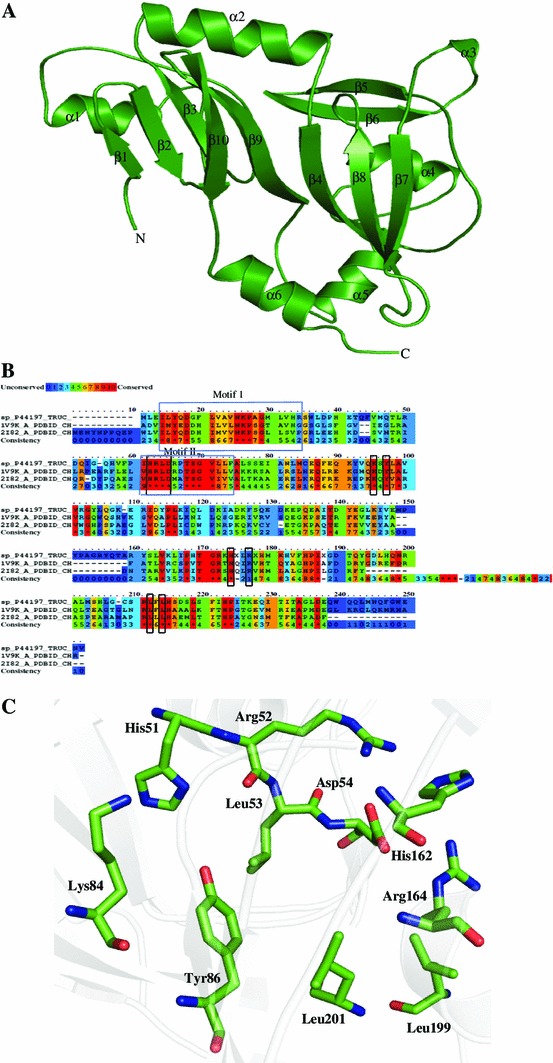
Representation of model structure of HP P44197. **a** Cartoon model showing overall structure. **b** Multiple sequence alignment showing conserved residues in *red color*, with a catalytic domain of P44197, Rlu C and *E. coli* Rlu A (PDB ID: 2I82) distinguished residues in *black box* and motifs I and II in *blue box*. **c** A detailed description of P44197 active site in which Asp54 is surrounded by arginine residues from both the sides namely Arg52 and Arg164

Ribosomal large subunit pseudouridine synthase C (PDB ID: 1V9K) is a lyase used as template for predicting the structure of HP. Ramachandran plots show 99.5 % of residues in allowed region. An rmsd of 0.379 Å^2^ with template was observed (Table 3). The predicted structure of P44197 is also an elongated, mixed α/β fold and possesses six α-helices and 10 β-strands (Fig. 3a), with an eight-stranded β sheet core. This structure is also a conserved Ψ synthase fold. Two sequence motifs (motif I and II) are present in the sequence of P44197 (Fig. 3b) (Koonin 1996), where motif II is the part of the active site, and architectural role was played by motif I (Koonin 1996). The active site of P44197 includes amino acid residues His51, Arg52, Leu53, Asp54, Lys84, Tyr86, His162, Arg164, Leu199, and Leu201 (Fig. 2c), in which Asp54 and Arg164 are conserved in the active site and mainly responsible for the catalytic activity (Del Campo et al. 2004).

The structure of HP P44197 was further analyzed using ProFunc and Dali servers. This analysis revealed a close similarity to the ribosomal large subunit pseudouridine synthase C (*Z* score = 34.5), ribosomal large subunit pseudouridine synthase D, (*Z* score = 26.7), etc. The best match showed 35 % sequence identity over aligned 217 residues. ProFunc server finds five pseudouridine synthase signature sequence, six ligand-binding templates and one DNA-binding template in the HP P44197. All these observations strongly suggest pseudouridylate synthase C activity in this HP (Table 3).

Similarly, RluC and RluD are homologous enzymes which convert three specific uridine bases to pseudouridine in *E. coli* ribosomal 23S RNA to pseudouridine, namely, bases 955, 2504, and 2580 in the case of RluC and 1911, 1915, and 1917 in the case of RluD and both contain N-terminal S4 RNA-binding domain (Mizutani et al. 2004). A loss of RluD which acts as an RNA chaperone is important for ribosome assembly. The loss of RluC results in reduced growth rate, and has no significant effect of impairing growth (Mizutani et al. 2004).

### HP P45267

The sequence analysis of HP P45267 showed that it is localized in the cytoplasm and is a non-secretary protein (Table S3). This protein showed a significant sequence similarity to the adenylate cyclase, which was further confirmed by domain analysis which suggested the presence of adenylate cyclase-like domain. The cluster analysis also suggests that HP P45267 belongs to the cluster of protein involved in the cAMP biosynthetic process. Analyzing SVMProt suggests that the HP P45267 is a member of zinc-binding protein and belongs to the DNA-binding protein family. Motif search showed the presence of CYTH-like phosphatases and adenylate cyclase motifs (Table 2). This is a virulent protein, which may be involved in the cellular process. The interacting partners of HP P45267 are Hsf-like protein, phosphatase, glutamate-ammonia-ligase, adenylyl transferase, DNA repair protein, outer membrane protein P2 (Figure S1). All these findings strongly suggest that the HP P45267 possesses CYTH-like adenylate cyclase activity and play significant roles in the survival of *H. influenzae,* and may be considered as a potential drug target (Carbonetti 2010).

**Fig. 4:**
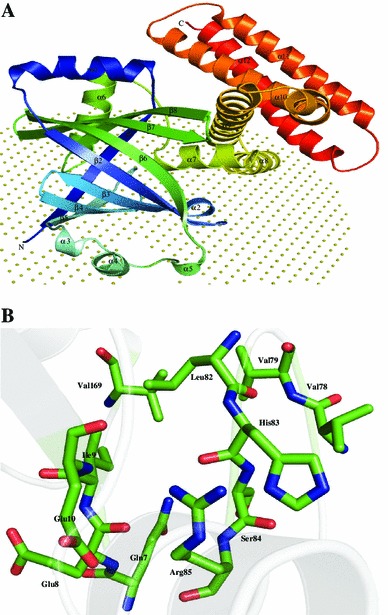
Representation of model structure of HP P45267. **a** Cartoon diagram showing N-terminal part is shown in *blue* while C-terminal in *red*. Collection of non-bonded spheres represents membrane. **b** Stick representation of P45267 active site with Glu10 residue is proposed to be involve in cAMP binding

Structure of P45267 was predicted by fold recognition algorithm on the sparksx server using adenylate cyclase (PDB ID: 2GFG) as template. PROCHECK reports that 98.8 % of residues are present in the allowed region of the Ramachandran plot. Overall structure contains 12 α-helices and 8 β-strands. Interestingly, eight stranded anti-parallel barrels (β1–β8) surrounded by nine long α-helices and three small helical regions fold were adopted by this protein (Fig. 4a) which looks like a cup or bottle with helical handles on both sides. The structure analysis shows that residues Met1, Val79, Pro108 and Phe109 are attached to the membrane. We found three AMP-binding residues Glu10, Arg62 and Arg127 in the P45267 (Figure S3). The active site may contain residues Gln7, Glu8, Ile9, Glu10, Val78, Val79, Leu82, His83, Ser84, Arg85, and Val169 (Fig. 4b).

The structure analysis results showed the presence of adenylate activity in this HP due to the high structural resemblance with the CYTH-like phosphatase (*Z* score = 34.5), adenylate cyclase 2 (*Z* score = 13.5), etc. We also found a similar search in ProFunc analysis, which shows the presence of 3 CYTH-like phosphatase motifs in this HP. These observations suggest that the HP P45267 may be a CYTH-like phosphatase and adenylate cyclase enzyme, and it is essential for pathogenesis (Table 3).

Adenylyl cyclase (AC, EC 4.6.1.1) catalyzes the cyclization of ATP to form cyclic AMP (cAMP), and is an important signaling molecule present in most of cells (Taussig and Gilman 1995). The liver cells produce cAMP, which is responsible for phosphorylase activation in response to extracellular adrenaline. It is found that adenylyl cyclase is involved in a wide variety of signal transduction mechanisms in different cell types (Sutherland 1972). In prokaryotes, expression of metabolic pathways such as the lactose operon, which are otherwise inactive when glucose is abundant, are triggered by cAMP (Black et al. 1980), and in eukaryotes, cAMP is involved in hormonal signal transduction or transmits stimulus from extracellular receptors to activate cell-specific mechanisms (Sutherland 1972). Danchin (1993) identified six distinct non-homologous classes of AC.

### HP Q57498

We obtained variable results for sub-cellular localization of HP Q57498. PSLpred (Bhasin et al. 2005) analysis revealed that this protein is localized in the periplasmic region. However, CELLO (Yu et al. 2006) tool suggests that it is a cytoplasmic protein. This is a non-secreted protein and shows the absence of transmembrane helix (Table S3). Similarity search shows a significant similarity to the alkyl hydroperoxidase (AhpD) core protein and carboxymuconolactone decarboxylase (CMD). Domain annotation and cluster analysis further confirmed that this protein belongs to a CMD family protein. Motif discovery shows that this HP contains a typical CMD motif, which was further confirmed by a motif search on MEME suite (Table 2). This HP is found to be a virulent protein on the basis VirulentPred. This structural information of this protein may be utilized for drug design and discovery. This HP interacts with transcriptional regulator, autonomous glycyl radical cofactor GrcA, dihydrolipoamide dehydrogenase, and type III restriction–modification system endonuclease-like protein. This indicates the functional significance of HP Q57498 (Figure S1).

**Fig. 5:**
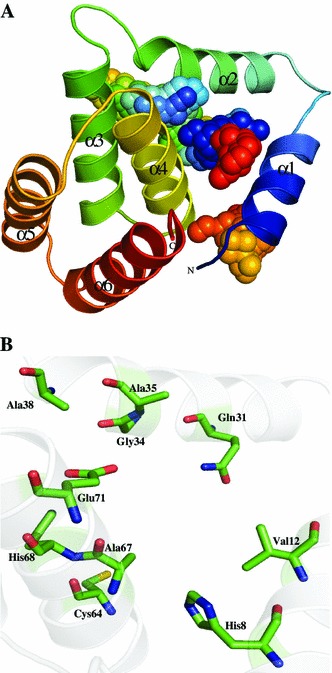
Representation of model structure of HP Q57498. **a** Cartoon model showing overall structure with active site residues. **b** A description of active site with His68 residue represents the citrate-binding site

Sparksx server (Yang et al. 2011) modeled the sequence of HP Q57498 where CMD proteins (PDB ID: 1VKE and 2QEU) were used as a template and 99 % residues in allowed region of the Ramachandran plot. The Q57498 predicted structure is showing all α-helix topology (six α-helices), with active cavities present between α1, α2 and α4 (Fig. 5a). We found citrate-binding sites in Q57498 which include residues corresponding Tyr30 and His68 (Figure S4). His68 is also present in the predicted active cavity along with His8, Val12, Gln31, Gly34, Ala35, Ala38, Cys64, Ala67, His68, and Glu71 (Fig. 5b). The ProFunc and Dali server searches, further used to validate the predicted function for Q57498, indicate the CMD activity in the HP. The Dali search mainly includes gamma-CMD (*Z* score = 11.6), CMD family protein (*Z* score = 10.7), etc., which are in top hits, showing the overall rmsd range of 0.7–3.1 Å^2^ over 97–133 residues. ProFunc validated the Dali results and showed the presence of five CMD and AhpD-like sequence motifs. ProFunc and Dali servers have also confirmed the structural similarity of HP Q57498 with that of CMD (Table 3).

The 3-oxoadipate pathway branches, namely catechol and protocatechuate, cause degradation of aromatic compounds. This pathway is important for the bacteria, unites at the common intermediate 3-oxoadipate enol-lactone, and the enzyme, CMD is involved in protocatechuate catabolism and gene fusion event leads to expression of CMD in bacteria (Eulberg et al. 1998).

### HP P44095

The HP P44095 is localized in the cytoplasm with no signal peptide and transmembrane helix (Table S3). Similarity search and sequence analysis show that HP P44095 is highly similar to the protein of cyclase family and metal-dependent hydrolase with cyclase activity (Table S2). Furthermore, cluster analysis shows that this protein belongs to the cyclase activity proteins containing clusters. Motif analysis tools have further predicted a putative cyclase motif and glucose transporter type 3 (GLUT3) signatures, respectively, in the HP P44095. All these analyses led to a conclusion that HP P44095 presumably works as a protein with cyclase activity. String analysis suggests various proteins such as gluconate permease, 3-hydroxyisobutyrate dehydrogenase, putative aldolase, and glycerol-3-phosphate regulon as a functional networking partner of the HP P44095 (Figure S1).

**Fig. 6:**
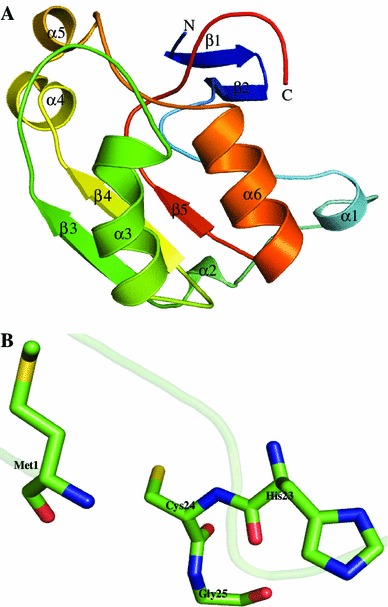
Representation of model structure of HP P44095. **a** Overall structure of P44095 shown in cartoon model with N-terminal is shown in *blue* and C-terminal in *red*. **b** Representation of the active site residues of P44095 in stick model

We used I-TASSER structure prediction server to compute the structure of P44095 using metal-dependent hydrolase with cyclase activity (PDB ID: 1R61) and manganese-dependent isatin hydrolase (PDB ID: 4J0N) as templates. A final model was validated with PROCHECK which shows that 98.3 % residues are in the allowed region of the Ramachandran plot. P44095 adopts mixed α/β fold with five β-strands and six α-helices (Fig. 6a). Residues Glu57, Pro60, Pro74, Tyr75, Ala76, Ala77, and Ile78 are responsible for membrane attachment in P44095. The structure and firestar server, COACH and COFACTOR analyses show the presence of three zinc-binding sites present at His23, His27, and Asp29 (Figure S5). Pocket-Finder predicts the active site residues as Met1, His23, Cys24 and Gly25 (Fig. 6b). We further analyzed this HP using ProFunc and Dali servers. Results showed a close structural similarity to the hydrolase (*Z* score = 13.9) and alpha amylase (*Z* score = 3.2). Furthermore, ProFunc found three cyclase motifs. ProFunc and Dali servers suggest that HP P44095 belongs to the cyclase family and possesses metal-dependent hydrolase activity (Table S4).

### HP P44093

HP P44093 shows cytoplasmic localization devoid of any transmembrane helix as well as the signal peptide, hence it may not be secreted (Table S3). This protein shows high sequence similarity to the 4-hydroxy-3-methyl but-2-enyl diphosphatereductase. We are unable to find any significant hit in most of the searches performed in various databases. However, SUPERFAMILY (Gough et al. 2001) shows that HP P44093 is a member of YgbK-like family, and PANTHER (Mi et al. 2005) predicts that this HP belongs to D-tagatose-1,6-bisphosphate aldolase family. This protein belongs to the cluster of proteins with ygbK-like activity. The classification system SVMProt (Cai et al. 2003) search shows that this HP belongs to the carbon–carbon lyases family. Motif finding tools suggest the presence of D-tagatose-1,6-bisphosphate aldolase motif and glycosyl hydrolases family 1 active site motifs in the HP P44093. Interaction partners of HP P44093 are aldolase, 3-hydroxyisobutyrate dehydrogenase, glycerol-3-phosphate regulon repressor, gluconate permease, l-fuculose phosphate aldolase, and d-xylose transporter subunit XylF indicating this protein plays a significant role in the carbohydrate metabolism (Figure S1).

**Fig. 7:**
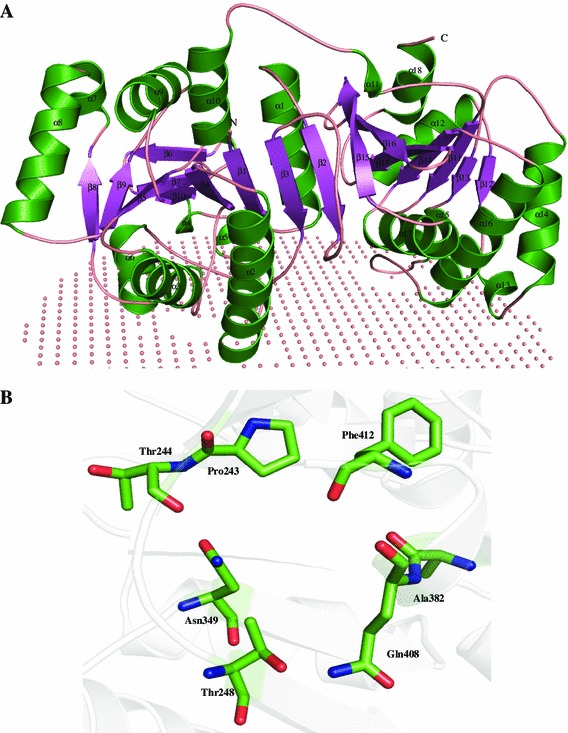
Representation of model structure of HP P44093. **a** Three-dimensional structure represented in cartoon model with membrane represented as non-bonded spheres. **b** Representation of the active site residues of P44093 in stick model

We used RaptorX sever for predicting the structure of P44093 using putative tRNA synthase (PDB ID: 3DQQ) as a template (Table 3) and found that 99.7 % residues in allowed region of the Ramachandran plot. The structure of P44093 contains 17 β-strands and 18 α-helices, and attains α/β fold with N-terminus covering one end and the C-terminus the other in a mixed parallel and anti-parallel fold (Fig. 7a). The residues Pro54, Asn56, Lys90, Ile281, Leu315, Ile318, Gln319, His320, Gln321, and Phe322 are membrane-attaching residues in P44093 (Fig. 7b). The firestar server was unable to detect any ligand-binding sites in P44093, while COACH and COFACTOR consensus show Ile7, Asn8 and Ile10 may be magnesium- and beta-d-glucose-binding sites. The P44093 also shows a conserved dihydroxyacetone phosphate-binding site (Figure S6), and the amino acid residues Pro243, Thr244, Thr248, Asn349, Trp382, Ala408, Gln409 and Phe412 may form the active cavity of the protein as suggested by the Pocket-Finder. Results of ProFunc and Dali servers show a close resemblance to the formimidoylglutamase (*Z* score = 7.2) and arginase-1 (*Z* score = 7.2) with overall rmsd of 1.5–4.0 Å^2^. Furthermore, three motifs with D-tagatose-1,6-bisphosphate aldolase were predicted in the sequence of HP P44093. Function assignment by ProFunc and Dali servers confirms the previously annotated results that the given HP is a D-tagatose-1,6-bisphosphate aldolase and tRNA synthase, respectively (Table S4).

It is known that tagatose-1,6-bisphosphate aldolase (TBPA), a tetrameric class II aldolase, catalyzes the reversible condensation of dihydroxyacetone phosphate with glyceraldehyde 3-phosphate to produce tagatose 1,6-bisphosphate. A comparison of TBPA with related fructose-1,6-bisphosphate aldolase (FBPA) shows common features associated with the mechanism. Major products of these enzymes catalyzed reactions differ in the chirality at a single position (Hall et al. 2002).

### HP P44720

Sub-cellular localization prediction with Psortb and PSLpred shows that HP P44720 is localized in cytoplasm, while CELLO suggests a periplasmic localization. Similarity search for HP P44720 shows high sequence similarity to aminodeoxychorismate lyase (Table S1 and S2). SUPERFAMILY shows that this HP is a member of zinc finger protein 425-like domain family. The cluster analysis using SYSTERS and ProtoNet shows that the protein belongs to the cluster of proteins with aminodeoxychorismate lyase activity. The motif discovery suggested the presence of zinc finger C2H2-type/integrase DNA-binding domain motif and aminodeoxychorismate lyase active site motif, respectively. The interaction partners of HP P44720 are DNA polymerase III subunit delta, thymidylate kinase, β-hexosaminidase, protease, Holliday junction resolvase-like protein, 3-oxoacyl-(acyl carrier protein) synthase I, acyl carrier protein indicating this protein plays a significant role in the pathogen metabolic system (Figure S1).

**Fig. 8:**
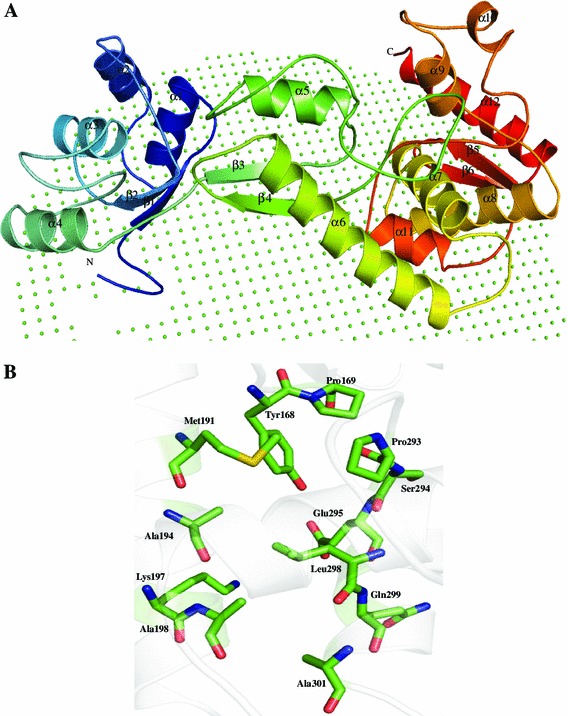
Representation of model structure of HP P44720. **a** Cartoon model representation of overall structure in which membrane is represented by non-bonded atoms. **b** Representation of the active site residues of P44720 in stick model

Structure of P44720 was predicted using amino deoxychorismate lyase protein (PDB ID: 2R1F) as a template (Table 3). The energy minimized model shows 99.7 % residues in the allowed region of the Ramachandran plot. The structure of P44720 shows six β-strands and 12 α-helices, and it assumes α/β fold with N-terminus covering one end and the C-terminus the other in a parallel fold (Fig. 8a). The membrane-attached residues in P44720 is Lys2, Phe4, Leu5, Ile6, Ala7 and Ile8 (Fig. 8b). Since Lys and Glu residues are important for catalytic activity of aminodeoxychorismate lyase (Nakai et al. 2000), we assume that residues corresponding to Tyr168, Pro169, Met191, Lys197, Ala198, Pro293, Ser294, Glu295, Leu298, Gln299, and Ala302 may form the active site cavity as suggested by the Pocket-Finder. Both ProFunc and Dali server searches suggested the presence of amino deoxychorismate lyase in this HP. Moreover, ProFunc shows seven motifs in the HP P44720. Function allocation by ProFunc and Dali servers further confirms that given HP is an amino deoxychorismate lyase (Table S4).

Chorismate is important in the biosynthesis of many important aromatic products such as anthranilate, prephenate, *p*-aminobenzoate and *p*-hydroxybenzoate, since it acts as a branch point precursor for the intermediary metabolites in bacteria (Green and Nichols 1991). 4-amino-4-deoxychorismate (ADC), formed from chorismate by the action of the enzyme p-aminobenzoate synthase, encoded by pabA and pabB, the product of pabC, is amino deoxychorismate lyase (ADCL) with pyridoxal 5′-phosphate (PLP) as a cofactor, converts ADC to *p*-aminobenzoate and pyruvate (Nichols et al. 1989; Ye et al. 1990), and along with *p*-aminobenzoate synthase is a key enzyme in the biosynthesis of *p*-aminobenzoate.

## Conclusions

Our study combines a number of bioinformatics tools for function predictions of previously not assigned proteins in the *H. influenzae* genome. We stressed that besides sequence analysis, structure can be used as a framework to explain known functional properties. Here we have combined the latest versions of several protein databases, protein motifs, features from the amino acid sequence, structure prediction, structure analysis, structure–function relationship, as well as a pathway and genome context methods to assign a precise function to hypothetical proteins for which no any experimental information is available. We used the sequence and structure-based methods for functional annotation of HPs from *H. influenzae* with lyase activity. The structure of these enzymes was predicted by most of the available methods, but we chose those models with best quality. HPs P44717, P44782, P44197, P45267, Q57498 and P44720 are categorized as lyase enzyme which was further confirmed by structure prediction and analysis. We also observed the reliability of different methods for predicting the three-dimensional structure of HPs and found that homology and fold recognition methods were only applicable to highly evolutionary conserved proteins and ab initio servers, especially I-TASSER gave good result for P44095 which is not predicted by other methods very precisely. Our in silico approach may be combined with experimental methods and make a leading contribution in functional elucidation of the protein structures.

## Electronic supplementary material

Below is the link to the electronic supplementary material.

Supplementary material 1 (DOCX 101 kb)

Supplementary material 2 (DOCX 101 kb)

Supplementary material 3 (DOCX 16 kb)

Supplementary material 4 (DOCX 13 kb)

**Figure S1:** Predicted interaction network of eight HPs with lyase activity of *H. influenzae* using the STRING database. **Figure S2**: Multiple sequence alignment of **(A).** P44717 and Q7VKS4 show three metal binding sites at residues Asp, Thr and Glu. **(B).** P44717 and Q87DZ3 displaying metal binding sites at Asp residues. **(C).** P44717 and Q8EDE1 presenting AMP binding site at Val residue (corresponding sites are shown in black boxes). **Figure S3**: The corresponding multiple cAMP binding sites are shown in P45267 and Q7CH76 at Glu and Arg residues depicted in black containers using multiple sequence alignment. **Figure S4**: Showing equivalent citrate binding sites in Q57498 and Q13HH1 at Tyr and His residues in black cases, as produce by multiple sequence alignment. **Figure S5**: Showing the presence of metal binding sites in P44095 and P84132 using multiple sequence alignment, present at His and Asp residues in black frames. **Figure S6**: Highlighting the dihydroxyacetone phosphate binding site at residue Gly in P44093 and G2SCE3 in black rectangle using multiple sequence alignment. (PPTX 3731 kb)
