# Supplementary material for: Structure-based functional annotation of putative conserved proteins having lyase activity from Haemophilus influenzae
Source: 3 Biotech. 2014 Jun 17;5(3):317–36. doi: 10.1007/s13205-014-0231-z (PMC4434415; doi:10.1007/s13205-014-0231-z)
Supplement: Supplementary file 3 — Supplementary material 3 (DOCX 16 kb) [file 13205_2014_231_MOESM3_ESM.docx]

| **S.No.** | **UNIPROT ID** | **PSORT B** | **PSLpred** | **CELLO** | **Signal peptide** | **SecretomeP**  (Secretion) | **HMMtop** | **TMHMM** |
| --- | --- | --- | --- | --- | --- | --- | --- | --- |
|  | **P44717** | Cytoplasmic membrane | Inner membrane protein | Inner membrane | Yes | No | 4 TM helix | 4 TM helix |
|  | **P44782** | Cytoplasmic | Cytoplasmic protein | Cytoplasmic | No | No | No TM helix | No TM helix |
|  | **P44197** | Cytoplasmic | Cytoplasmic protein | Cytoplasmic | No | No | No TM helix | No TM helix |
|  | **P45267** | Cytoplasmic | Cytoplasmic protein | Cytoplasmic | No | No | No TM helix | No TM helix |
|  | **Q57498** | Unknown | Periplasmic protein | Cytoplasmic | No | No | No TM helix | No TM helix |
|  | **P44095** | Unknown | Cytoplasmic protein | Cytoplasmic | No | No | No TM helix | No TM helix |
|  | **P44093** | Unknown | Cytoplasmic protein | Periplasmic | No | No | No TM helix | No TM helix |
|  | **P44720** | Cytoplasmic | Cytoplasmic protein | Periplasmic | No | Yes | 1 TM helix | 1 TM helix |

**Table S3: List of predicted Subcellular localization of HP with lyase activity in *H. influenzae*strain Rd KW20**
