# Supplementary material for: Structure-based functional annotation of putative conserved proteins having lyase activity from Haemophilus influenzae
Source: 3 Biotech. 2014 Jun 17;5(3):317–36. doi: 10.1007/s13205-014-0231-z (PMC4434415; doi:10.1007/s13205-014-0231-z)
Supplement: Supplementary file 4 — Supplementary material 4 (DOCX 13 kb) [file 13205_2014_231_MOESM4_ESM.docx]

**Table S4:** List of predicted structure analysis results of HP with lyase activity *H. influenzae* strain Rd KW20

| **S. NO.** | **UNIPROT ID** | **^a^ HHPred result** | **^b^ ProFunc** | **^c^ DALI server** |
| --- | --- | --- | --- | --- |
|  | **P44717** | CBS domain associated CorC/HlyC transporter | CBS domain associated CorC/HlyC transporter | CBS domain proteins like hemolysin etc. |
|  | **P44782** | Ribosomal large subunit pseudouridine synthase A | Pseudouridylate synthase | Ribosomal large subunit pseudouridine synthase A |
|  | **P44197** | Ribosomal large subunit pseudouridine synthase C | Pseudouridine synthase | Ribosomal large subunit pseudouridine synthase C |
|  | **P45267** | CYTH-like phosphatase and Adenylate cyclase 2 | CYTH-like phosphatases, Adenylate cyclase | Adenylate cyclase |
|  | **Q57498** | Carboxymuconolactone decarboxylase family protein | Function-Carboxymuconolactone decarboxylase | Carboxymuconolactone decarboxylase family protein |
|  | **P44095** | Metal-dependent hydrolase with cyclase activity | Cyclase like protein | Metal-dependent hydrolase with cyclase activity |
|  | **P44093** | Putative tRNA synthase | D-tagatose-1,6-bisphosphate aldolase | Putative trna synthase/ Formimidoylglutamase |
|  | **P44720** | Predicted aminodeoxychorismate lyase | Predicted aminodeoxychorismate lyase | Aminodeoxychorismate lyase |

^a.^ <http://toolkit.tuebingen.mpg.de/hhpred>

^b.^ <http://www.ebi.ac.uk/thornton-srv/databases/ProFunc/>

^c.^ <http://ekhidna.biocenter.helsinki.fi/dali_lite/start>
