# Supplementary material for: Structure-based functional annotation of putative conserved proteins having lyase activity from Haemophilus influenzae
Source: 3 Biotech. 2014 Jun 17;5(3):317–36. doi: 10.1007/s13205-014-0231-z (PMC4434415; doi:10.1007/s13205-014-0231-z)
Supplement: Supplementary file 5 — Figure S1: Predicted interaction network of eight HPs with lyase activity of H. influenzae using the STRING database. Figure S2: Multiple sequence alignment of (A). P44717 and Q7VKS4 show three metal binding sites at residues Asp, Thr and Glu. (B). P44717 and Q87DZ3 displaying metal binding sites at Asp residues. (C). P44717 and Q8EDE1 presenting AMP binding site at Val residue (corresponding sites are shown in black boxes). Figure S3: The corresponding multiple cAMP binding sites are shown in P45267 and Q7CH76 at Glu and Arg residues depicted in black containers using multiple sequence alignment. Figure S4: Showing equivalent citrate binding sites in Q57498 and Q13HH1 at Tyr and His residues in black cases, as produce by multiple sequence alignment. Figure S5: Showing the presence of metal binding sites in P44095 and P84132 using multiple sequence alignment, present at His and Asp residues in black frames. Figure S6: Highlighting the dihydroxyacetone phosphate binding site at residue Gly in P44093 and G2SCE3 in black rectangle using multiple sequence alignment. (PPTX 3731 kb) [file 13205_2014_231_MOESM5_ESM.pptx]

## Slide 1
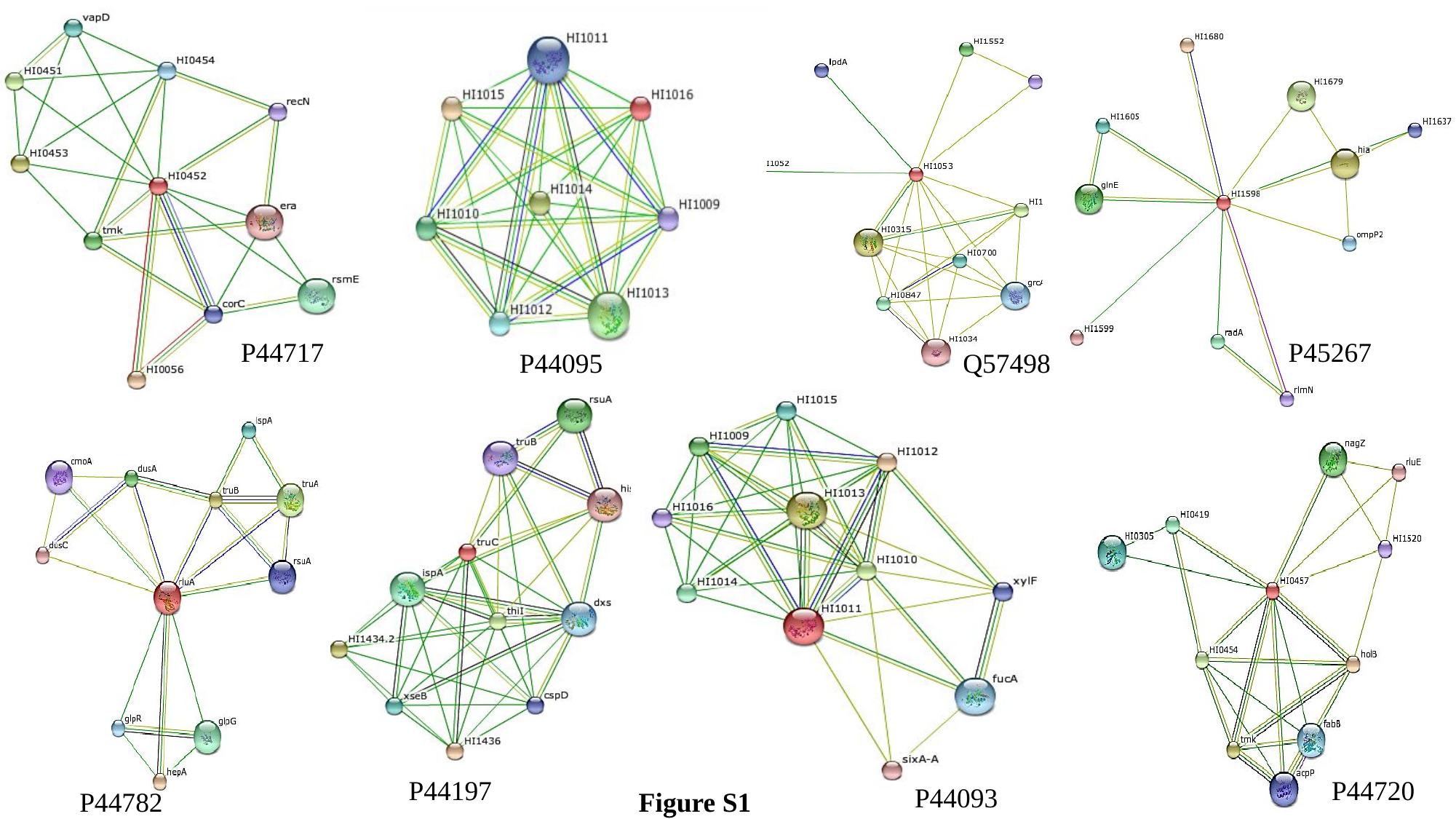

P44717
P45267
P44095
Q57498
P44197
P44720
P44093
P44782
Figure S1

## Slide 2
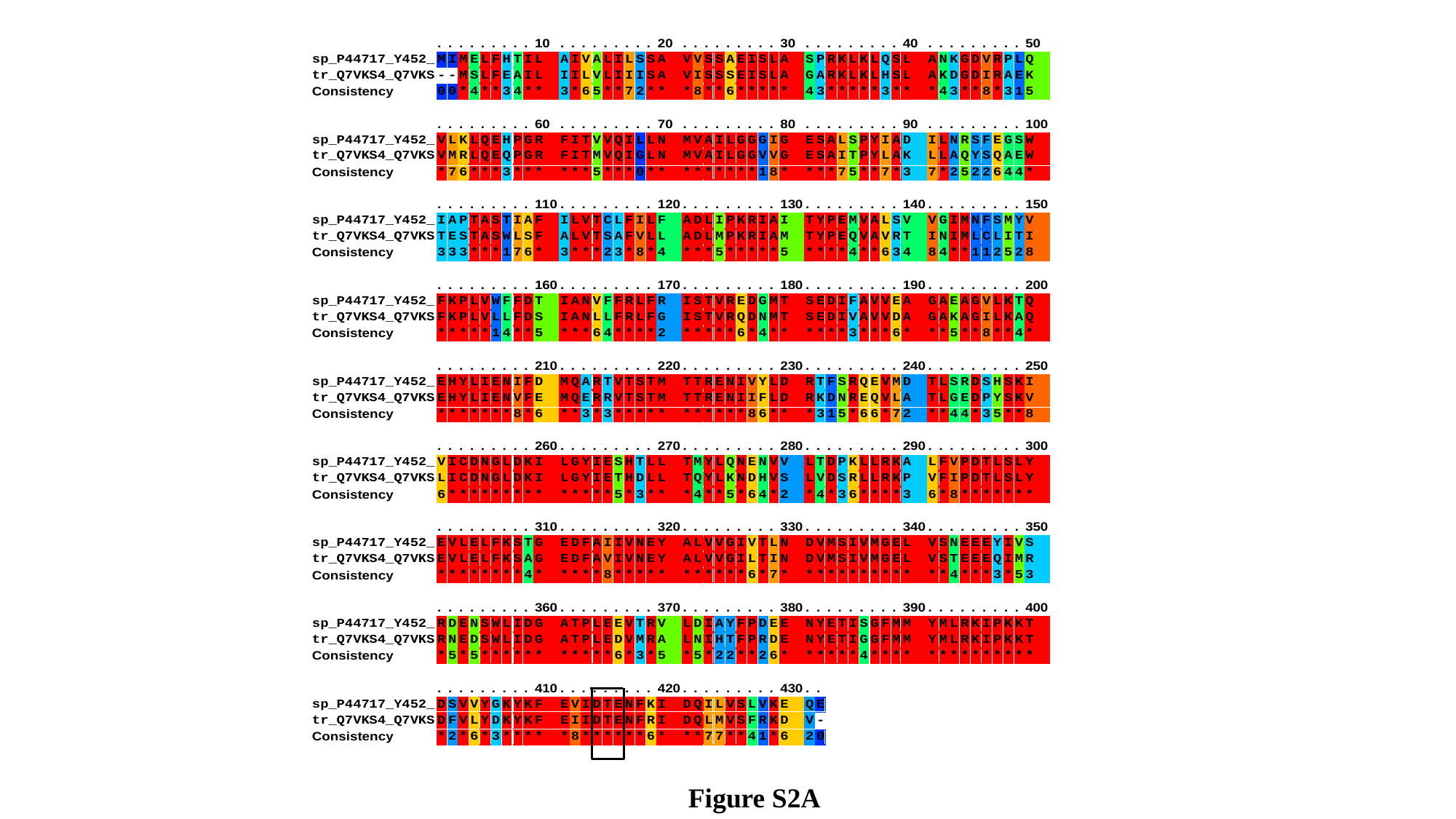

Figure S2A

## Slide 3
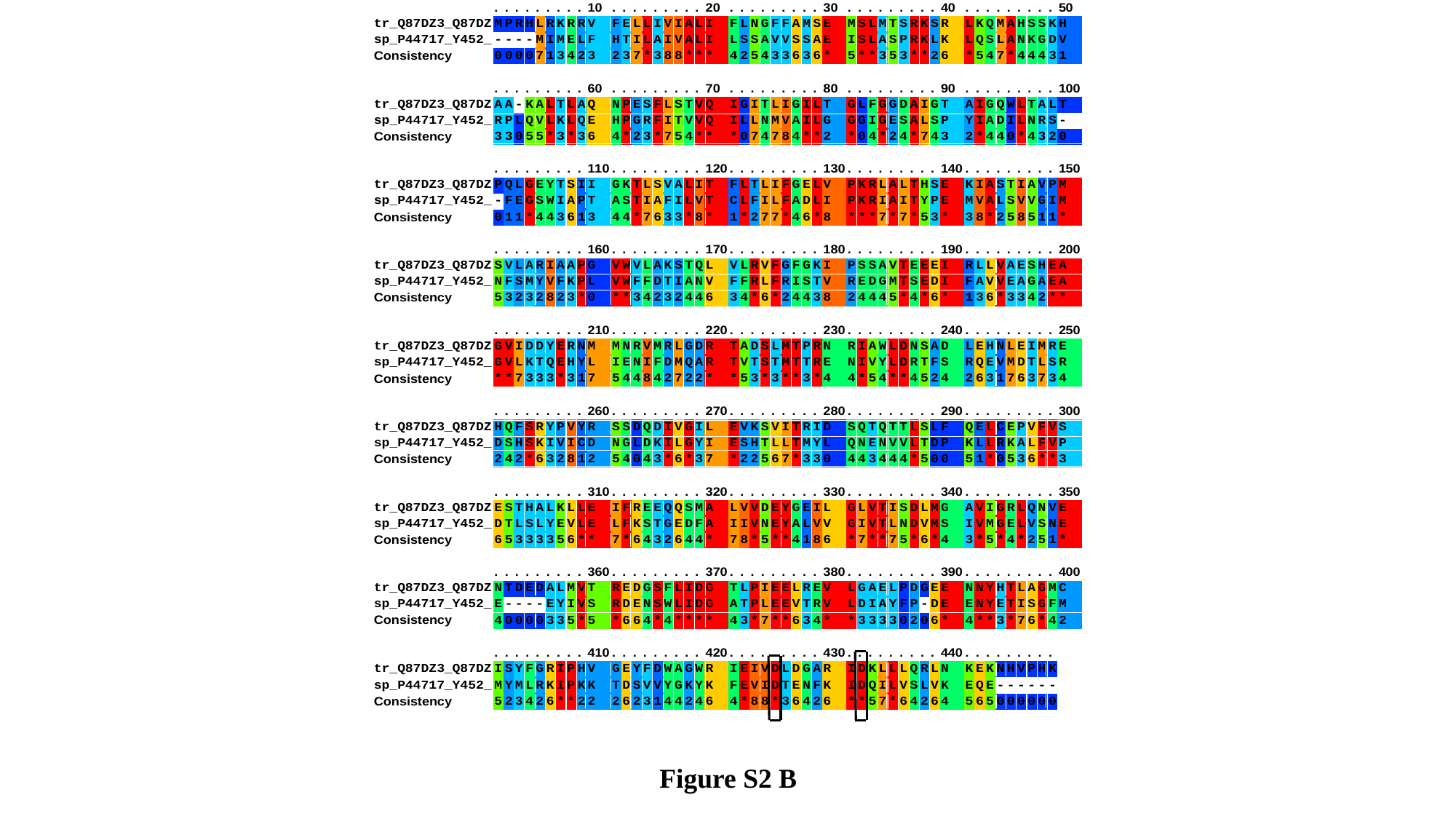

Figure S2 B

## Slide 4
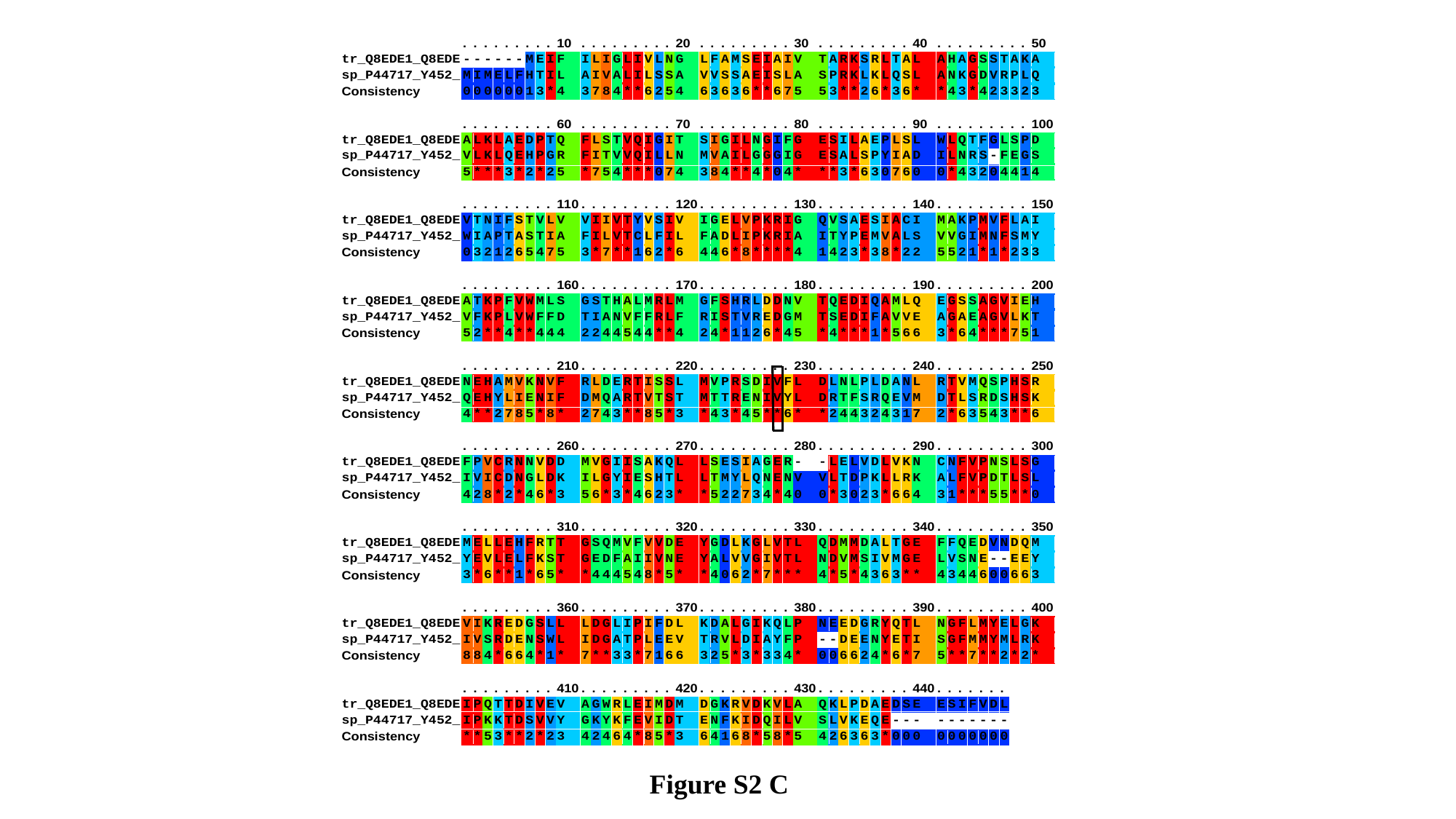

Figure S2 C

## Slide 5
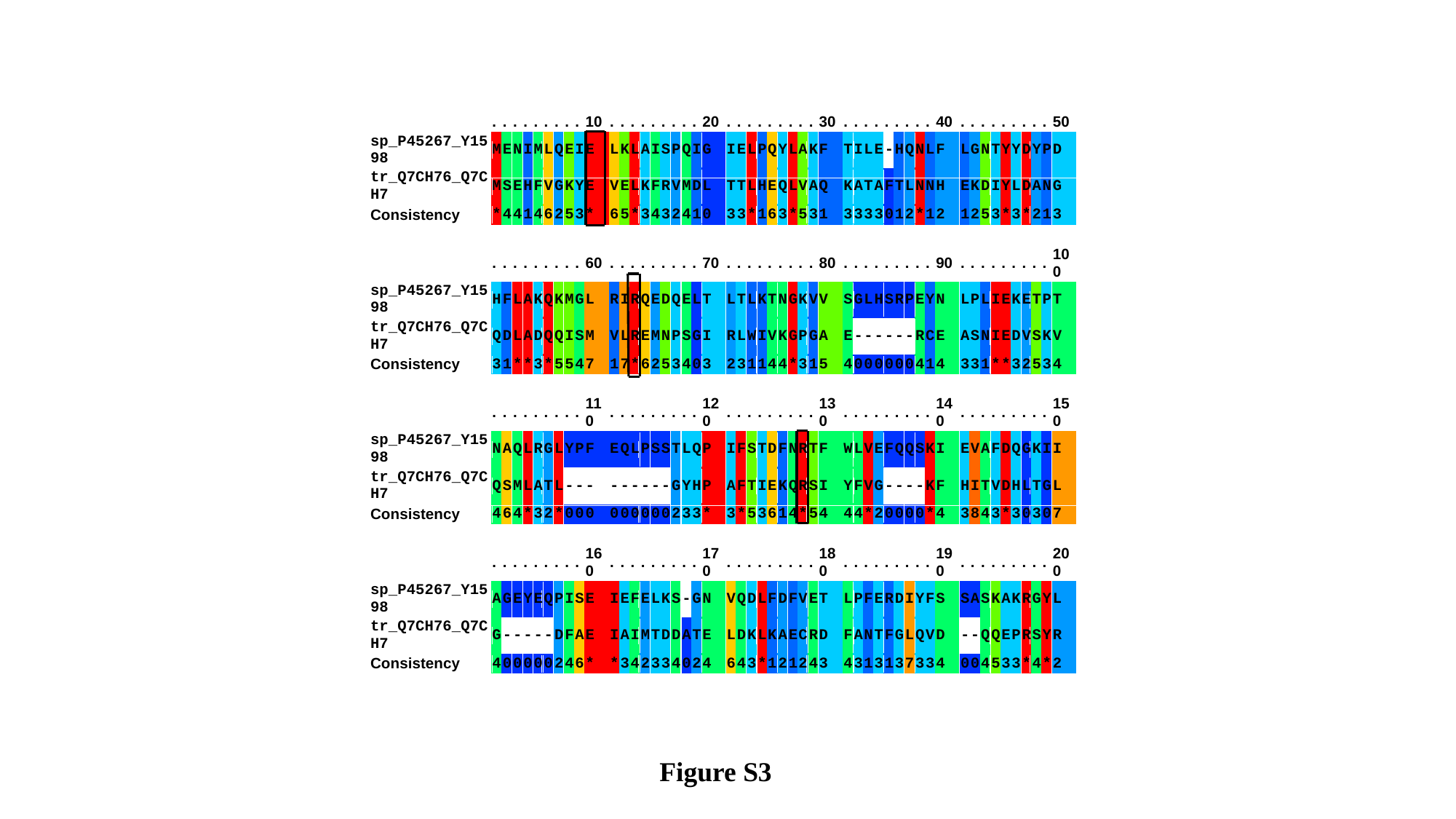

Figure S3

## Slide 6
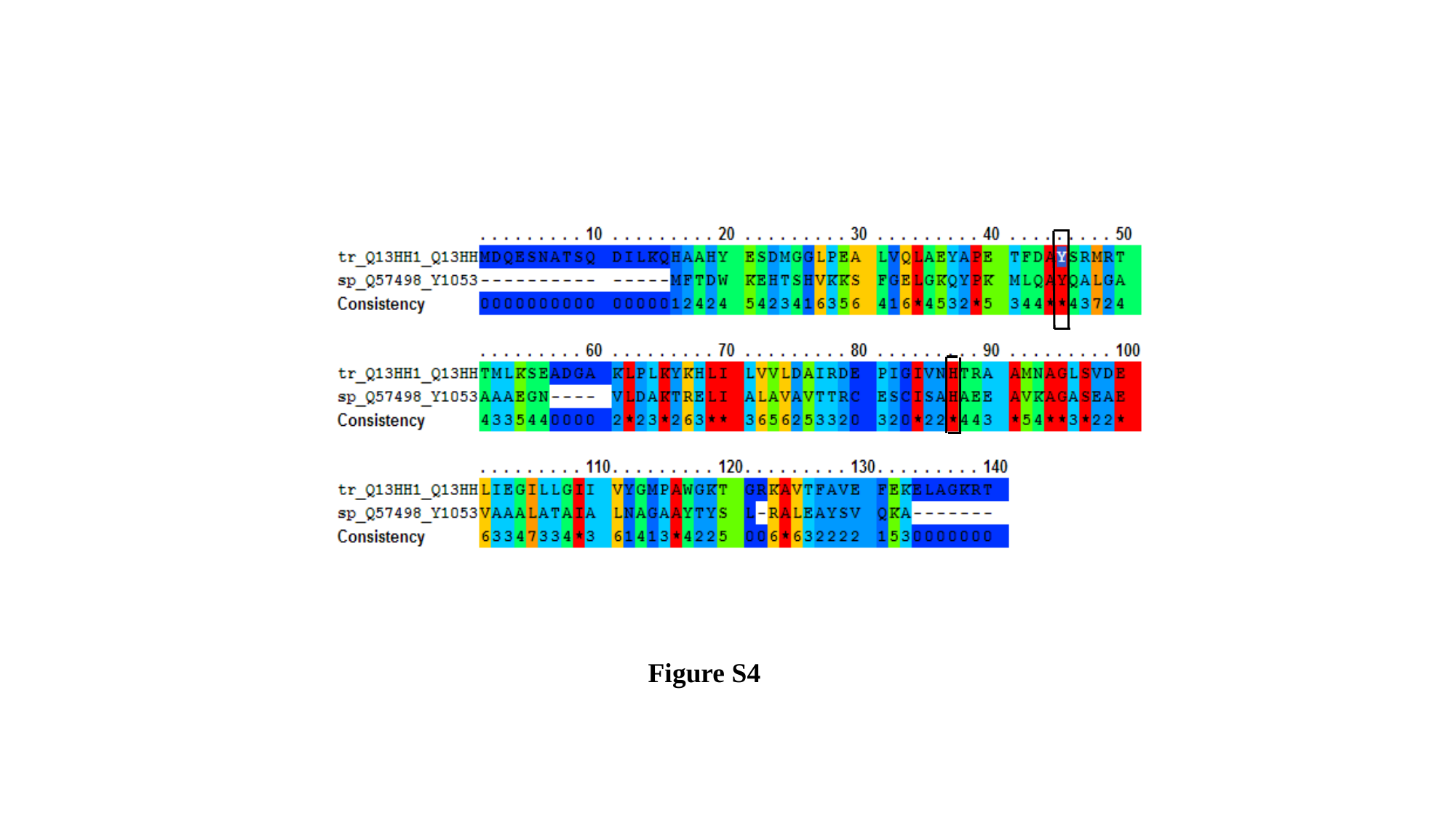

Figure S4

## Slide 7
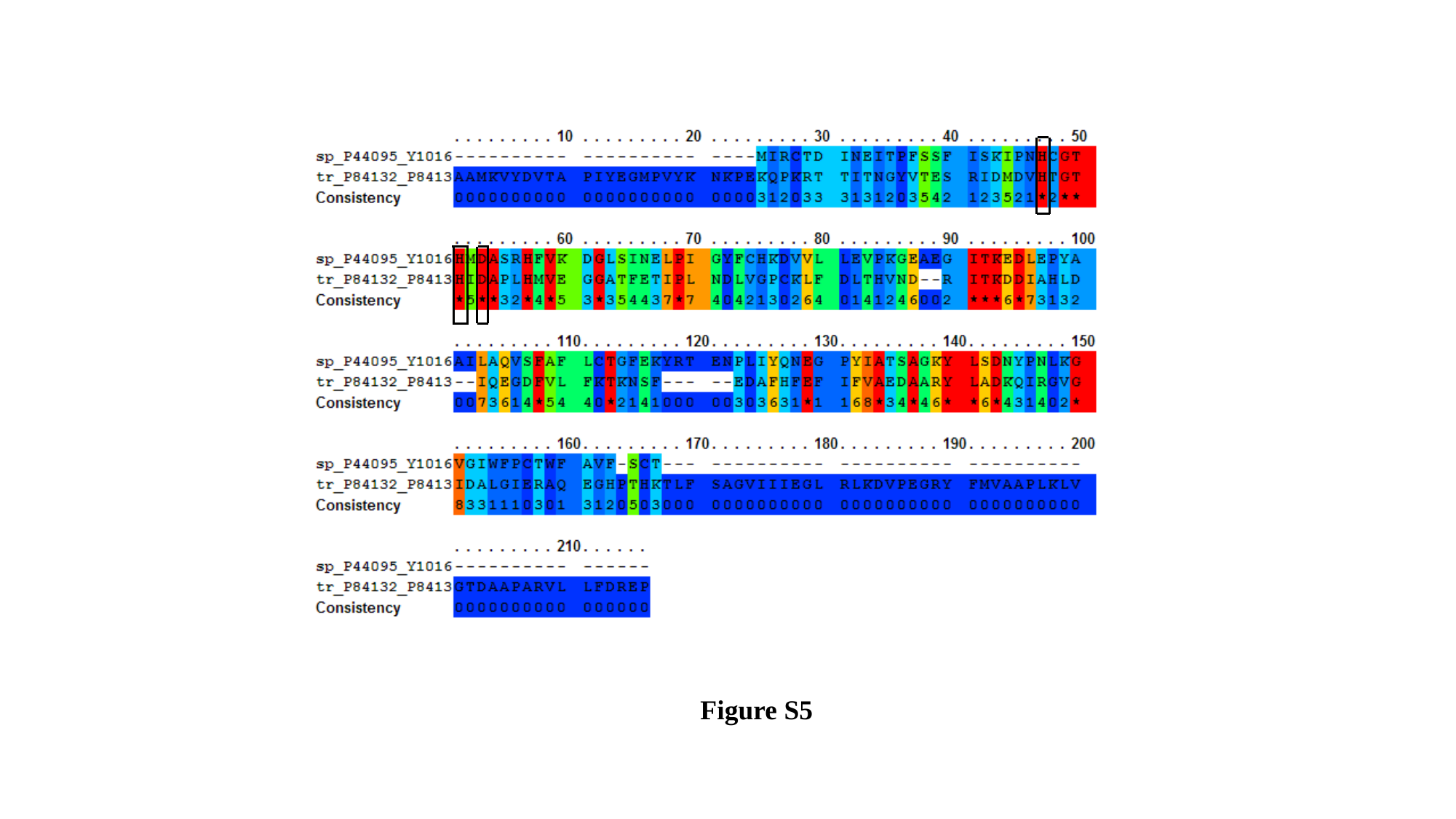

Figure S5

## Slide 8
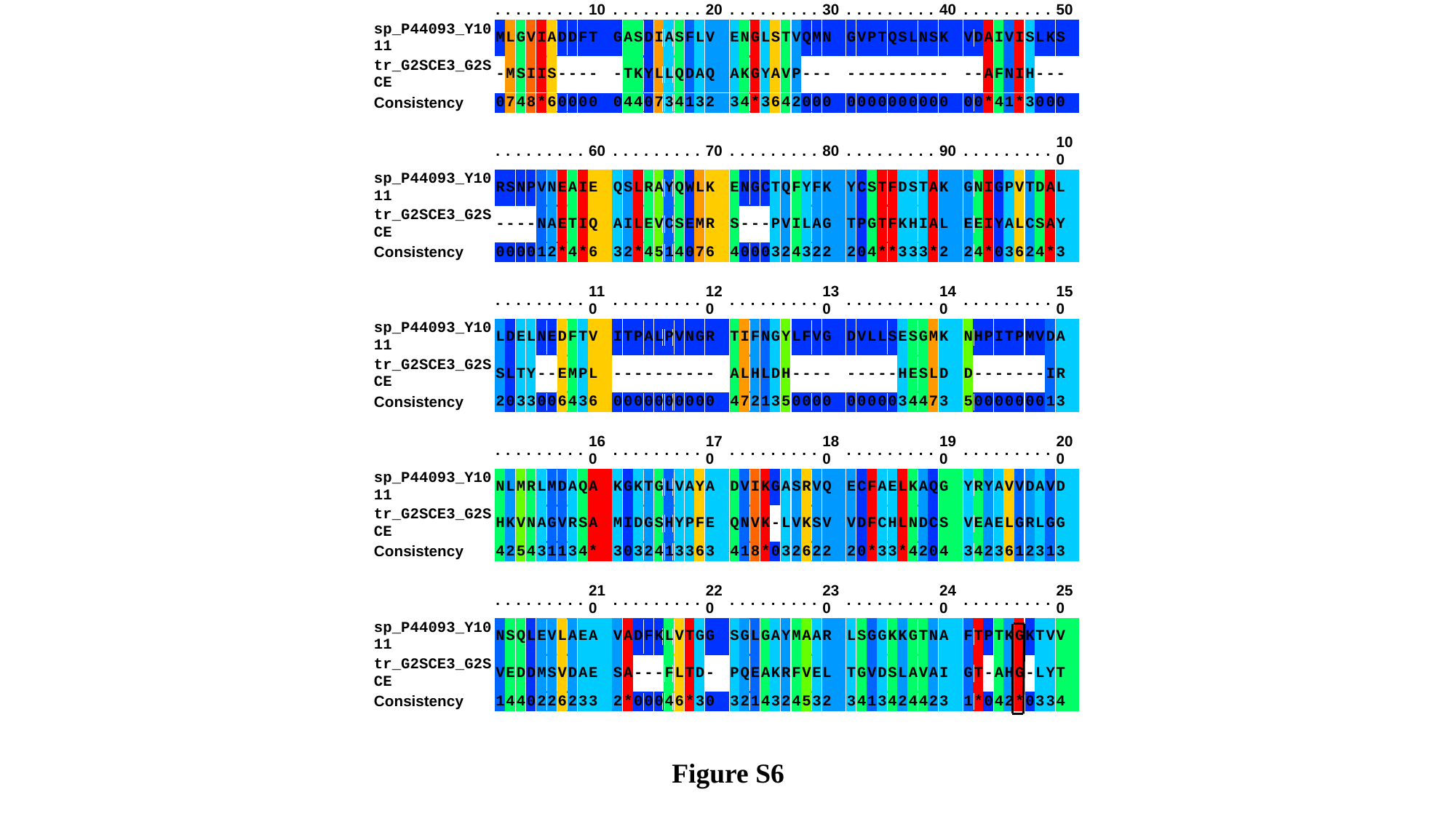

Figure S6
